# Comparative Effectiveness of Supervised and Home-Based Rehabilitation after Anterior Cruciate Ligament Reconstruction in Competitive Athletes

**DOI:** 10.3390/jcm13082245

**Published:** 2024-04-12

**Authors:** Rehan Iftikhar Bukhari Syed, Laszlo Rudolf Hangody, Gergely Frischmann, Petra Kós, Bence Kopper, István Berkes

**Affiliations:** 1 Doctoral School of Clinical Medicine, Semmelweis University, 1085 Budapest, Hungary; berkesdr@gmail.com; 2 Department of Traumatology, Semmelweis University, 1085 Budapest, Hungary; hangodylaszlo@gmail.com; 3 Biomechanics Lab, TSO Medical Hungary Kft., 1118 Budapest, Hungary; frischmann.gergely@tsomedical.com (G.F.); kos.petra@tsomedical.com (P.K.); 4 Department of Biomechanics, Hungarian University of Sports Science, 1123 Budapest, Hungary; kopper.tf@gmail.com; 5 Department of Health Sciences and Sport Medicine, Hungarian University of Sports Science, 1123 Budapest, Hungary

**Keywords:** anterior cruciate ligament, anterior cruciate ligament reconstruction, supervised rehabilitation, home-based rehabilitation, return to sport, prevention, re-injury

## Abstract

**Background:** After the increasingly common anterior cruciate ligament reconstruction (ACLR) procedure in competitive athletes, rehabilitation is crucial for facilitating a timely return to sports (RTS) and preventing re-injury. This pilot study investigates the patient-reported outcomes of postoperative rehabilitation in competitive athletes, comparing supervised rehabilitation (SVR) and home-based rehabilitation (HBR). **Methods:** After ACLR, 60 (out of 74 screened) athletes were recruited and equally divided into HBR and SVR groups using non-probability convenience sampling, with each group comprising 15 males and 15 females. The rehabilitation outcomes in the respective groups were evaluated at 8 months using measures (Tegner Activity Scale [TAS], International Knee Documentation Committee subjective knee form [IKDC-SKF], ACL Return to Sport after Injury [ACL-RSI]) and objective parameters (isometric muscle strength, hamstring/quadricep asymmetry). RTS was evaluated at 9 months, with ACL re-injury rates recorded approximately 6 months post-RTS. **Results:** Both groups exhibited decreased TAS scores (HBR: 8 to 6, SVR: 8 to 7), with the SVR group demonstrating superior postoperative IKDC-SKF scores (81.82 vs. 68.43) and lower ACL-RSI scores (49.46 vs. 55.25). Isometric and isokinetic muscle strength, along with asymmetry values, was higher in the SVR group 8 months post-ACLR (*p* < 0.05). The SVR group showed a higher RTS rate to the same level (76.6% vs. 53.3%), while the re-injury rate was the same in both the rehabilitation groups (3.3%). **Conclusions:** Although both rehabilitation approaches yielded comparable outcomes, SVR may demonstrate some superior biomechanical improvements in athletes, resulting in a higher RTS rate. However, the psychological outcomes and re-injury rates did not significantly differ between the groups, emphasizing the need to address individual psychological needs during rehabilitation. Further investigation is recommended with a larger sample size to address the differences of gender among competitive athletes.

## 1. Introduction

Anterior cruciate ligament (ACL) injuries are among the most common competitive athletic injuries resulting in musculoskeletal and neurophysiologic dysfunctions [1,2]. ACL reconstruction (ACLR) is considered the best treatment of choice after an ACL injury in athletes, with the aim to restore the stability, strength, and functional ability of the ACL-deficient knee, thus making a safe return to sports (RTS) possible [3]. Rehabilitation is considered as an integral component of the treatment process after ACLR in athletes, because it can dramatically affect the final outcome of the surgery [4]. Postoperative rehabilitation can be conducted either at a specialized rehabilitation clinic, referred to as supervised rehabilitation (SVR), or in the athlete’s home, known as home-based rehabilitation (HBR). The comparative effectiveness of these rehabilitation methods following ACLR in athletes has been a topic of considerable debate [5,6]. This study investigates whether SVR yields superior outcomes compared to HBR in competitive athletes post-ACLR. However, recent research findings have not consistently supported the notion that SVR leads to better postoperative results than HBR [5]. While some studies suggest that HBR can be effective, particularly in the early stages post-ACLR [7,8], others have highlighted additional benefits associated with SVR, such as vocational training to enhance participant confidence [9]. Notably, Rhim H.C. and colleagues concluded that SVR offers advantages in terms of improved muscular strength, neuromuscular control, and self-reported knee function scores compared to HBR after ACLR [10]. Despite these findings, there remains a gap in the literature regarding the psychological outcomes associated with SVR and HBR in competitive athletes. A comprehensive evaluation, incorporating both subjective and objective measures, is essential for assessing psychological readiness for a safe RTS [11].

The literature has reported high success rates of ACLR in competitive athletes [12], but fewer than half of them can resume their competitive sport participation and regain their pre-injury level of functional capabilities after ACLR [13,14]. RTS constitutes a crucial milestone in the rehabilitation process following ACLR in competitive athletes. However, there is currently a lack of evidence specifying the criteria for progression or discharge in this context [15,16]. Quadricep and hamstring strength deficits, an abnormal hamstring–quadricep ratio (H-Q ratio), and the lack of motivation are the main risk factors related to re-injury [17,18,19]. These risk factors can be addressed using outcome measures [20]. Psychological readiness is crucial for a safe RTS after ACLR [21], yet its assessment and the subsequent support provided during rehabilitation are not well documented [22,23].

Therefore, our study aims to evaluate the effectiveness of SVR versus HBR in preventing re-injury post-ACLR among competitive athletes, considering both biomechanical and psychological outcomes. Specifically, we hypothesize that SVR would lead to superior outcomes compared to HBR in athletes after ACLR. By addressing this gap in the literature, our study contributes to a better understanding of the optimal rehabilitation approach for competitive athletes undergoing ACLR, ultimately facilitating a safer and more successful RTS.

## 2. Materials and Methods

### 2.1. Design

This pilot study was conducted at Castle Park Surgical Hospital (Tata, Hungary) and TSO Biomechanics Lab (Budapest, Hungary) between January 2020 and February 2023. It was ethically approved by the Regional and Institutional Science and Research Ethic Committee of Semmelweis University, Budapest, Hungary (SE RKEB number: 120/2021). Prior written informed consent was obtained from all the patients. The study was designed as a non-randomized observational study without blinding.

### 2.2. Patient Enrollment

**Figure 1:**
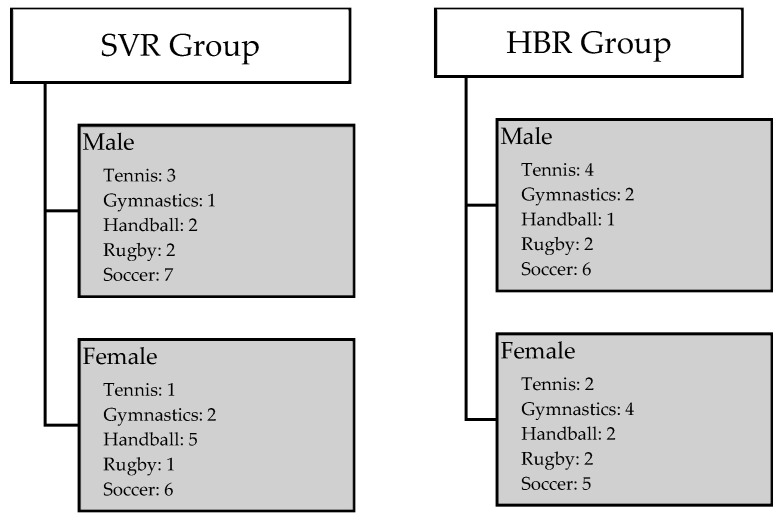
Type of sport: A gender-differentiated breakdown of sports participation among athletes in SVR and HBR groups.

Participants were enrolled based on specific criteria without random allocation to treatment groups, and there was no blinding of participants or researchers involved in the study. A predetermined sampling methodology was implemented based on predefined inclusion and exclusion criteria in order to ensure systematic and transparent enrollment. Competitive athletes involved in high-risk pivoting sports, specifically soccer, rugby, handball, gymnastics, and tennis, and diagnosed with non-acute isolated ACL injuries underwent surgical reconstruction. All the surgeries were performed by a single operating surgeon at Castle Park Surgical Hospital in Tata, Hungary, between January 2020 and March 2021. Selection criteria, aligned with the American College of Cardiology [24], included competitive athletes of both genders, aged 15 to 50 years, with diagnosed non-acute isolated ACL injuries and no secondary underlying pathology, having undergone ACLR. Exclusion criteria comprised non-competitive athletes, individuals below 15 or above 50 years of age, and those with multiple ligamentous or bony injuries and secondary underlying pathologies. To provide a comprehensive understanding of the athletic activities undertaken by participants, we presented a detailed gender-differentiated breakdown of types of sports, as shown in Figure 1. Following ACLR, 74 patients underwent screening based on the inclusion criteria, of whom 14 were excluded due to unresolved medical complications, concurrent injuries, or lack of consent, ensuring the recruitment of eligible participants. Ultimately, 60 participants were recruited and divided into two equal groups using non-probability convenience sampling as follows: 30 in the SVR group and 30 in the HBR group (each group comprising 15 males and 15 females). The SVR group was considered as the case while the HBR group was considered to be a control group.

### 2.3. Surgical Technique

ACLR was performed using the arthroscopic transtibial technique. Patients were placed in the supine position, and a thigh tourniquet was applied. Initial visualization was achieved through a standard anterolateral portal, followed by diagnostic arthroscopy to assess ACL injury extent and associated pathologies. Graft harvesting involved quadruple-bundle hamstring (semitendinosus and gracilis) autografts. Tendons were harvested and prepared for reconstruction. Femoral tunnel creation utilized a transtibial approach. A guide pin was inserted through the tibial tunnel to establish the femoral tunnel footprint, followed by careful reaming for optimal graft placement.

During surgery, attention was given to addressing meniscus injuries. Tears were repaired using inside-out or all-inside techniques when feasible. Otherwise, a partial meniscectomy was performed to enhance joint stability. Graft fixation involved an endobutton on the femoral side and Milagro^®^ advance interference absorbable screws (DePuy Synthes, Johnson & Johnson) on the tibial side. Graft tensioning was meticulously conducted to ensure stability.

### 2.4. Rehabilitation Protocols

**Figure 2:**
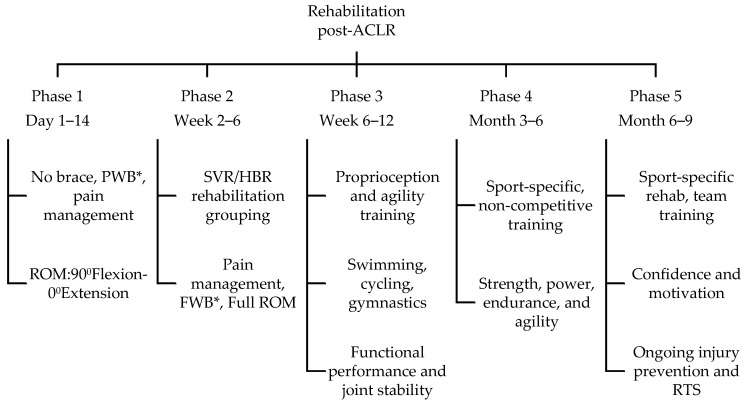
Rehabilitation flow chart. The flow chart represents the phase-wise division of rehabilitation process post-ACLR in 5 distinct phases. * Partial weight bearing (PWB) and full weight bearing (FWB) are indicated.

Following ACLR, a structured rehabilitation program was implemented to optimize the recovery process and facilitate a safe RTS. The rehabilitation protocol consisted of five distinct phases, with a focus on pain management, mobility, and range of motion (ROM) improvement in the initial phases, while the later phases emphasized improvements in the strength, power, endurance, stability, and extensibility of the associated knee structures [23], as illustrated in Figure 2.

During the initial 8 months post-surgery, participants’ visits to the physiotherapist ranged from 40 to 64 for the SVR group and 5 to 12 for the HBR group. The SVR participants attended supervised physical therapy classes twice a week in an outpatient program in a rehabilitation clinic, with each session lasting 90–120 min. The classes predominantly focused on proprioceptive and functional training exercises, with periodic updates [25].

In contrast, participants in the HBR group performed all the remedial exercises unsupervised at home. They were provided with written instructions and pictorial representations of the exercises to be performed, with a recommended minimum of two exercise sessions per week. Adherence to the treatment plan was monitored through periodic assessments at the rehabilitation clinic, where adjustments, education, and modifications were provided as necessary. Patient adherence was reinforced through regular communication channels, including follow-up appointments and remote consultations, with the frequency and progression of exercises tailored to individual responses under the discretion of the treating therapist. Monitoring methods included in-person clinic visits and remote consultations via phone or video calls.

In the HBR group, the participants maintained exercise logs to track their home exercise sessions, reviewed during clinic visits for adherence and progress adjustments. In the SVR group, while there were no prescribed home exercises, their attendance at and participation in supervised physical therapy were monitored during clinic visits. Efforts were made to standardize the exercise protocols across both groups, with adjustments made based on individual responses. Although not utilized, the potential benefits of smartphone apps for monitoring adherence were acknowledged for future research.

Both rehabilitation groups underwent five mandatory follow-up examinations, including the removal of stitches at postoperative day 14, followed by the division into the SVR and HBR groups. Phase 1 was identical for both groups. The pain management protocol during phase 2 primarily utilized non-pharmacological techniques such as cryotherapy, manual therapy, and neuromuscular techniques to alleviate discomfort. At the 6-week mark, a second examination assessed activity level progression. Phases 2–5 were carried out with same exercise programs either supervised or home-based. A third review at 3 months evaluated patients’ ability to perform complex physical activities actively, while a fourth review at 6 months assessed progress towards achieving the physical attributes necessary for patients’ RTS. The intensity and frequency of training were standardized for all patients, with the only difference being supervision [26].

At the 8-month mark, a fifth follow-up examination was conducted, subjecting all the participants to subjective and objective evaluations at the Biomechanics Lab. Based on these evaluations, the patients were permitted to gradually participate in their respective competitive sports only if they met certain criteria, which were mainly designed by their treating therapist, following scientific literature guidelines [27,28]. The mean time period to RTS after ACLR in both rehabilitation groups was approximately 9 months, and re-injury rates were measured and recorded at the 5–6-month mark following the RTS. The rehabilitation protocol was based on “Campbell’s Operative Orthopedics” textbook [29].

### 2.5. Outcome Measures

The Tegner Activity Scale (TAS) score was used to assess the participants according to their level of sports activity, using a numeric scale of 0 to 10, where 0 represented knee-related disability and 10 represented the highest level of competitive sport.

The International Knee Documentation Committee subjective knee form (IKDC-SKF) was utilized to subjectively evaluate knee functional scores using a self-reported scale ranging from 0 (lowest knee function) to 100 (highest knee function). This patient-completed tool contains sections on knee symptoms (7 items), function (2 items), and sports activities (2 items), with scores ranging from 0 points (lowest level of function or highest level of symptoms) to 100 points (highest level of function and lowest level of symptoms).

Psychological readiness to RTS was evaluated using the ACL Return to Sport after Injury (ACL-RSI) questionnaire, via which the athlete’s subjective responses were recorded using 12 structurally designed questions. The ACL-RSI questionnaire, developed by Webster, Feller, and Lambros (2008) [23], comprises a total of 12 questions regarding emotional wellbeing (5 questions), the level of confidence in performing the respective sport (5 questions), and risk appraisal (2 questions). The percentage of the total score indicates the psychological response of a patient.

### 2.6. Assessment of Muscle Strength and Neuromuscular Control

The isometric maximum strength of the quadricep and hamstring muscles was measured in kilograms using Kinvent Isometric Dynamometers (KINVENT, France; K-Pull and K-Push Handheld Dynamometers) at knee flexion angles of 30°, 45°, and 90°. The percentage of strength deficits in the muscles of the operated side was compared to the non-operated side at each specific angle. For all three measured joint angles, the quadricep and hamstring ratio was calculated (H/Q ratio). To assess the quadricep concentric contractions, we utilized the Kineoglobus system (Kinetic Systems Inc., Boston, MA, USA). These measurements were conducted during knee joint extension at angular velocities of 120° and 240°.

Static and dynamic balance (standing and unilateral squatting) tests were performed on KINVENT Force Plates and the average COP (Center of Pressure) position was measured. The differences in average foot pressures (%) were calculated between the sides during both measurements.

The maximum isometric strength of the hip adductors and abductors was measured on a Vald Performance Force Frame at a knee joint angle of 60 degrees. The results were calculated in Newtons and the force deficit between the sides and the agonist–antagonist ratio were also calculated.

Re-injury was detected through clinical and MRI examinations performed by the operating doctor. RTS was recorded according to the athlete’s reported sports participation.

### 2.7. Statistical Analysis

To assess the adequacy of our sample size for detecting meaningful differences in the measured endpoints, we conducted a post hoc power analysis after data collection. Given the lack of available data for performing an a priori power calculation, we utilized GPower software (version 3.1.9.7) to calculate the post hoc power based on the observed effect sizes and sample sizes obtained from our study.

Data are represented by averages and standard deviations. To define the adequate statistical procedure, a Shapiro–Wilk W test was performed to identify normality. For the comparison of the datasets, we used a combination of statistical tests. Specifically, we utilized paired sample *t*-tests or Wilcoxon tests for within-group comparisons, and independent sample *t*-tests or Mann–Whitney U tests for between-group comparisons. For the discrete value comparisons, a Chi-Squared test was calculated. JASP (version 0.17.1) and Statistica (version 14.0.1) statistical software (TIBCO Statsoft USA) were used for the calculations. The significance level was set at *p* < 0.05.

To provide additional information about the samples included in the manuscript, we included the effect size (Cohen’s d) and the post hoc power values for cases where significant differences (*p* < 0.05) were detected. The power values were calculated using GPower software.

## 3. Results

### 3.1. Baseline Characteristics

In this study, a total of 60 participants were included, with a deliberate and equal allocation of 15 male and 15 female individuals to each of the SVR and HBR groups. This gender-balanced distribution was methodologically chosen to enhance the robustness of our analysis, aiming to mitigate potential gender-related biases. This decision was not driven by the specific frequency of ACL injuries but rather by the strategic objective of ensuring a representative cohort for comprehensive assessment. The mean age in the SVR group was 22.43 ± 6.34 years, while in the HBR group, it was 24.96 ± 7.93 years. However, the difference in age between the two groups was not statistically significant (*p* = 0.1991). Likewise, there were no significant differences in height and weight between the groups, with mean values of 174.78 ± 9.59 cm and 172 ± 9.81 cm for height and 71.11 ± 12.90 kg and 77.23 ± 20.41 kg for weight in the SVR and HBR groups, respectively (*p* = 0.3022 for height, *p* = 0.1960 for weight). Lastly, the mean follow-up time was 8.62 ± 7.32 months for the SVR group and 8.48 ± 7.68 months for the HBR group, with no significant difference between the two groups (*p* = 0.9501). These results indicate that, at baseline, both rehabilitation groups were comparable in terms of gender distribution, age, height, weight, and follow-up time.

A *t*-test for BMI was calculated separately for men and women. No significant difference in BMI was found among the male and female athletes in the respective rehabilitation groups, indicating that the groups were comparable. Table 1 displays the demographic data for the participants in the study.

### 3.2. Patient-Reported Questionnaires

The TAS scores for the male participants in the SVR group decreased from 8 (preoperatively) to 7 (postoperative day (POD) 240), while in the HBR group, they decreased from 7 (preoperatively) to 5 (POD 240). Similarly, for female participants, the SVR group showed a decrease from 7 (preoperatively) to 6 (POD 240), whereas the HBR group displayed a decrease from 8 (preoperatively) to 6 (POD 240). The average TAS score in the SVR group was 8 preoperatively and 7 at POD 240, while in the HBR group, it was 8 preoperatively and 6 at POD 240. These results suggest that both rehabilitation approaches led to a reduction in TAS scores postoperatively. However, the average postoperative score was slightly lower in the HBR group compared to the SVR group.

Prior to surgery, the mean preoperative IKDC-SKF score in the SVR group was 49, compared to 45 in the HBR group. The results demonstrated a significant difference in IKDC-SKF scores at POD 240 between the SVR and HBR groups. The mean IKDC-SKF score at POD 240 in the SVR group was 81.82, compared to 68.43 in the HBR group (*p* = 0.0021). It is worth mentioning that the IKDC-SKF scores improved for both groups postoperatively.

Conversely, the mean ACL-RSI score at POD 240 in the HBR group was 55.25 ± 9.72, while it was 49.46 ± 8.14 in the SVR group (*p* = 0.0194). These findings indicate that individuals in the SVR group achieved higher IKDC-SKF scores, suggesting better postoperative outcomes. However, individuals in the HBR group had higher ACL-RSI scores, indicating a greater psychological readiness to RTS compared to the SVR group. Unfortunately, preoperative data for ACL-RSI scores were not available.

### 3.3. Comparison of Muscle Strength and Neuromuscular Control Parameters

At 30 degrees, the percentage of isometric strength deficit in the quadriceps between the operated and non-operated limb was 26.1% in the SVR group and 27.9% in the HBR group. Similarly, the percentage of isometric strength deficit in the hamstrings was 14.1% in the SVR group and 32.2% in the HBR group. The percentage of H/Q asymmetry at 30 degrees was 10.9% in the SVR group and 1.1% in the HBR group, and that was not significant for either comparison (Table 2).

At 45 degrees, the percentage of isometric strength deficit in the quadriceps between the operated and non-operated limb was 22.3% in the SVR group and 22.1% in the HBR group. Similarly, the percentage of isometric strength deficit in the hamstrings was 12.8% in the SVR group and 47.8% in the HBR group. The percentage of H/Q asymmetry at 45 degrees was not significant in the SVR group (0.8%) but was significant in the HBR group (16.6%, *p* < 0.05).

At 90 degrees, the percentage of isometric strength deficit in the quadriceps between the operated and non-operated limb was 23.1% in the SVR group and 23.9% in the HBR group. The percentage of isometric strength deficit in the hamstrings was 69.7% in the SVR group and 84.9% in the HBR group. The percentage of H/Q asymmetry at 90 degrees was significant in both the SVR group (37.9%) and the HBR group (30.5%, *p* < 0.05). The results of our power analysis revealed that our study design had achieved satisfactory power levels across the measured endpoints, indicating that our sample size was adequate to detect meaningful differences if they existed.

No significant differences were observed in the percentages of weight distribution deficit in stance evaluation, squat analysis, and hip abductor and adductor force and asymmetry measurements. These findings suggest that both rehabilitation approaches led to similar outcomes in terms of these variables, as shown in Table 3.

### 3.4. Return to Sport

In the SVR group, 76.6% of individuals were able to return to the same level of sport participation following ACL rehabilitation. Additionally, 16.6% were able to return to a lower level of sport participation, while 6.6% did not return to any sport activities. In contrast, the HBR group had a lower percentage of individuals returning to the same level of sport participation, with only 53.3% achieving this outcome. Furthermore, 30% of the individuals in this group were able to return to a lower level of sport participation, while 16.6% did not return to any sport activities. The observed disparities in sport participation levels between the two groups are substantiated by the data presented in Table 4, where a Chi-Squared test contingency table indicated a significant difference (*p* = 0.036).

### 3.5. ACL Re-Injury

The comprehensive evaluation of ACL re-injury rates necessitates a subtle understanding of the distinction between re-injury to the previously operated knee and new injuries, particularly those affecting the contralateral knee. In both the SVR and HBR groups, the overall re-injury rate was 3.3%. However, it is crucial to delineate the contralateral ACL injury rate, which was 6.6% in the SVR group and 3.3% in the HBR group.

## 4. Discussion

A recent systematic review concluded that previous studies had failed to demonstrate a significant difference between SVR and HBR [5]. This lack of differentiation stems primarily from inadequate assessment of patient-reported outcomes, particularly among elite athletes. Our study compared the outcomes of SVR versus HBR in competitive athletes after ACLR, considering both biomechanical and psychological outcomes. The comparison of our findings with the literature elucidates key insights. Our deliberate gender-balanced allocation aimed to enhance the study’s robustness and minimize gender-related biases. While not directly aligned with the literature, this methodology aligns with the overarching goal of creating a representative cohort for comprehensive assessment [12]. The comparable baseline characteristics, including age, height, weight, and follow-up time, corroborate previous studies emphasizing the importance of homogeneous cohorts for accurate evaluation [12,13,14]. 

Our study recognized the importance of investigating gender differences in ACL rehabilitation outcomes. While both male and female participants were included, the initial analysis lacked detailed gender-specific insights. A deeper understanding of gender influences in ACL rehabilitation is imperative, urging future research to delve into the underlying mechanisms and devise targeted interventions to address these disparities. Our study’s observed reduction in TAS scores postoperatively for both rehabilitation groups concurs with the literature, indicating a commonality in the impact of rehabilitation on activity levels [12,13]. The slightly lower average postoperative TAS score in the HBR group aligns with findings suggesting variations in activity scale outcomes based on rehabilitation methods [6]. This trend prompts further investigation into the factors influencing postoperative activity levels and highlights the need for tailored rehabilitation approaches.

While our results align with the literature indicating a significant improvement in IKDC-SKF scores postoperatively for both rehabilitation groups [12], it is noteworthy that SVR yielded superior postoperative outcomes in this regard. This finding may be attributed to the more structured and closely monitored nature of SVR, which likely resulted in better adherence to the rehabilitation protocol and more efficient recovery progress. Conversely, HBR participants exhibited greater psychological readiness for an RTS, potentially due to differences in patient perceptions, coping mechanisms, resilience, and reduced fear of re-injury. Our findings support the existing literature emphasizing the importance of addressing psychological factors during rehabilitation to optimize outcomes [21,22,23]. Integrating consultations with sports psychologists and utilizing interventions targeting psychological factors in athletes could further enhance the rehabilitation process and promote a successful RTS [30]. Psychic responses generally improve during rehabilitation, but in some cases, fear may increase and become a serious risk factor when returning to sport [31]. RTS is not just significantly influenced by normal postoperative knee function [32]. Additionally, individuals with lower levels of optimism may particularly benefit from targeted interventions aimed at enhancing psychological readiness for RTS [33].

Muscle strength imbalances are of particular concern in individuals after ACLR. The knee flexion angles at 30°, 45°, and 90° were chosen for their biomechanical relevance during functional activities, ensuring consistency with the existing literature and providing valuable insights into muscle performance relevant to knee stability. Significant differences in the dynamometric values favoring the SVR group in muscle strength and symmetry values highlight its greater efficiency in rehabilitation, consistent with the literature indicating potential benefits of supervised programs [6]. Further research is warranted to explore the underlying mechanisms contributing to the observed H/Q asymmetry at different degrees of knee flexion and to optimize rehabilitation strategies for improving muscle balance across a wider range of motion.

The significant divergence of RTS percentages between the rehabilitation groups echoes the literature emphasizing the impact of rehabilitation methods on RTS [13,14]. Our study showed an overall re-injury rate of 3.3%, consistent with the literature emphasizing the importance of considering different types of injuries [16]. The observed contralateral ACL injury rates indicate that, while the overall re-injury rate is consistent between the two groups, the distribution of injuries differs. This insight provides a more refined perspective on the nature of injuries and enhances the interpretation of rehabilitation outcomes in the context of contralateral ACL lesions. Monitoring re-injury rates 5–6 months after resuming sports activities provided valuable insights into the effectiveness of rehabilitation protocols in preventing further injuries. These thoughtfully chosen time points aimed to capture critical recovery milestones and evaluate the associated risks and outcomes involved in resuming sporting activities. Successful ACL restoration involves unrestricted sports participation and a return to pre-injury levels. Considering the influence of fear of re-injury is crucial in assessing ACLR outcomes [34], SVR may provide athletes with more challenging training, especially in the later phases of rehabilitation, in such a way that they can develop their sport-specific skills and expertise more confidently and comfortably [35]. 

The selected time periods in this study were strategically determined to assess key aspects of postoperative recovery. The evaluation at eight months post-operation provided a comprehensive assessment of muscle strength and knee function, reflecting a substantial recovery period. At nine months post-operation, the focus shifted to evaluating the capacity to return to sport. Optimizing recovery after ACL reconstruction requires comprehensive rehabilitation plans that prioritize the restoration of muscular strength and functional status in both the reconstructed knee and the unaffected limb, as emphasized in the literature [36]. The literature indicates that criterion-based rehabilitation after ACLR is essential to enable effective recovery and allow athletes to achieve their RTS goals while extenuating impairments related to re-injury [37,38]. In addition to the fulfillment of these objective criteria, a rehabilitation program should also focus on improving the subjective knee functional and psychological readiness scores [39]. A prerequisite for a safe and early RTS is to take into account the latest evidence concerning re-injury prevention and to establish ongoing professional communication between the injured athlete, the coach, the physician, and the physiotherapist [40]. To achieve the best recovery outcomes for competitive athletes, it is imperative to prioritize the psychological readiness of athletes in the supervised group.

## 5. Limitations

The study’s reliance on a single surgeon may limit the generalizability of our findings. Future research involving multiple surgeons and institutions is essential to validate our results and ensure broader applicability to diverse patient populations. Surgeon variability underscores the importance of multi-center studies to strengthen the reliability of conclusions regarding post-ACLR rehabilitation strategies.

Secondly, the decision not to conduct a gender-specific analysis due to the resulting reduction in sample sizes could be perceived as a limitation. While maintaining an equal gender distribution was essential, the absence of gender-specific analyses restricts our understanding of potential differences between male and female athletes. Our study did not adjust for multiple comparisons, like the Bonferroni correction. While aiding interpretability, this choice may heighten the risk of Type I error (false positives), potentially impacting result robustness. Consequently, we recognize this as a pilot study, laying the foundation for future investigations with larger sample sizes and separate analyses for male and female groups employing multiple comparisons.

Lastly, the absence of universally acknowledged isokinetic dynamometers for biomechanical measurements might be considered a limitation. However, it is essential to note that our primary objective was to compare various samples rather than establish comparisons with universally recognized datasets. Given the accuracy of the employed devices, we deemed simpler equipment sufficient to achieve our study objectives effectively.

## 6. Conclusions

Both rehabilitation approaches demonstrated comparable outcomes among competitive athletes post-ACLR. However, SVR provided some additional advantages in athletes by enhancing patient-reported outcomes, leading to a comparable rate of return to same-level sport. Nevertheless, there was no disparity in the re-injury rate, potentially due to a lack of notable improvements in psychological outcomes compared to the HBR group, especially after an average of 8 months following ACLR. Therefore, to achieve a successful RTS and prevent re-injury in competitive athletes, it is crucial to implement criterion-based rehabilitation programs. These programs should include continuous psychological preparation supervised by a physiotherapist. However, given the preliminary nature of our findings, further investigations should incorporate comprehensive psychological assessments beyond measures such as the ACL-RSI. Future research should explore the use of larger sample sizes, narrow age ranges, and long-term follow-up to comprehensively evaluate the effectiveness of rehabilitation in preventing re-injury after ACLR.

## Figures and Tables

**Table 1:** Demographic data of participants in the SVR and HBR groups. Values are expressed as mean ± standard deviation. The table describes the uniform distribution of baseline characteristics (gender, age, height, weight, follow-up time, and BMI in male and female participants) among the two rehabilitation groups, along with their significance values.

Baseline Characteristics	SVR (*n* = 30)	HBR (*n* = 30)	*p*
Gender (male/female)	15/15	15/15	
Age (years)	22.43 ± 6.34	24.96 ± 7.93	0.199
Height (cm)	174.78 ± 9.59	172 ± 9.81	0.302
Weight (kg)	71.11 ± 12.90	77.23 ± 20.41	0.196
Follow-up time (months)	8.62 ± 7.32	8.48 ± 7.68	0.950
BMI (Male)	22.19 ± 2.02	23.93 ± 2.75	0.070
BMI (Female)	24.23 ± 2.52	25.53 ± 3.66	0.327

**Table 2:** Comparison of various measured biomechanical data values between SVR and HBR groups. O represents operated, NO non-operated leg. Data are represented by means and standard deviations, asterisk (*) indicates significant difference between SVR and HBR groups (*p* < 0.05, independent sample *t*-test).

Measurement Procedure	Group	Mean	SD	*p*	Effect Size	Power
30 deg Max Isometric Quadricep Strength (kg) O	SVR	54.614	16.295	0.077		
	HBR	44.810	18.607			
30 deg Max Isometric Quadricep Strength (kg) NO	SVR	68.957	16.720	0.035 *	0.664	0.689
	HBR	57.323	18.257			
30 deg Quadricep Asymmetry (%)	SVR	21.519	11.708	0.969		
	HBR	21.352	15.479			
30 deg Max Isometric Hamstring Strength (kg) O	SVR	21.514	6.237	0.224		
	HBR	19.217	6.091			
30 deg Max Isometric Hamstring Strength (kg) NO	SVR	24.686	6.528	0.730		
	HBR	25.465	8.194			
30 deg Hamstring Asymmetry (%)	SVR	17.490	10.564	0.042 *	0.633	0.662
	HBR	24.970	12.832			
30 deg H/Q Ratio (%) O	SVR	38.462	11.329	0.218		
	HBR	43.979	16.704			
30 deg H/Q Ratio (%) NO	SVR	34.664	7.189	0.018 *	0.752	0.782
	HBR	43.412	14.672			
45 deg Max Isometric Quadricep Strength (kg) O	SVR	58.252	15.364	0.214		
	HBR	51.448	19.344			
45 deg Max Isometric Quadricep Strength (kg) NO	SVR	71.233	17.792	0.155		
	HBR	62.868	19.986			
45 deg Quadricep Asymmetry (%)	SVR	21.019	11.199	0.781		
	HBR	19.895	14.650			
45 deg Max Isometric Hamstring Strength (kg) O	SVR	20.233	6.684	0.031 *	0.673	0.691
	HBR	16.304	4.953			
45.deg Max Isometric Hamstring Strength (kg) NO	SVR	24.233	6.163	0.981		
	HBR	24.183	7.993			
45 deg Hamstring Asymmetry (%)	SVR	19.967	13.815	0.010 *	0.81	0.84
	HBR	31.696	15.057			
45 deg H/Q Ratio (%) O	SVR	34.025	10.749	0.786		
	HBR	33.066	11.922			
45 deg H/Q Ratio (%) NO	SVR	33.790	7.743	0.131		
	HBR	38.528	11.878			
Isokinetic Leg Extension 240° (Kg) O	SVR	20.850	6.716	0.007 *	1.183	0.885
	HBR	13.809	4.781			
Isokinetic Leg Extension 240° (Kg) NO	SVR	25.114	6.431	0.010 *	1.133	0.86
	HBR	17.664	6.763			

**Table 3:** Comparison of abductor, adductor force, asymmetry, stance, and squat weight distribution for the SVR and HBR groups. O represents operated, NO non-operated leg. Data are represented by means and standard deviations, no significant difference was detected between SVR and HBR groups (*p* < 0.05, independent samples *t*-test).

Measurement Procedure	Group	Mean	SD	*p*
Max Isometric Hip Adductor Strength at 60. Knee Flexion (N) O	SVR	379.837	96.169	0.164
	HBR	339.609	89.725	
Max Isometric Hip Adductor Strength at 60. Knee Flexion (N) NO	SVR	387.188	90.290	0.160
	HBR	348.174	88.023	
Hip Adductor Asymmetry (%)	SVR	5.633	3.356	0.956
	HBR	5.694	3.760	
Max Isometric Hip Abductor Strength at 60. Knee Flexion (N) O	SVR	354.967	87.474	0.127
	HBR	315.326	79.593	
Max Isometric Hip Abductor Strength at 60. Knee Flexion (N) NO	SVR	352.650	76.089	0.163
	HBR	318.630	80.340	
Hip Abductor Asymmetry (%)	SVR	7.759	5.368	0.854
	HBR	8.076	5.814	
Hip ABD/ADD Ratio (%) O	SVR	94.480	15.259	0.958
	HBR	94.752	18.359	
Hip ABD/ADD Ratio (%) NO	SVR	92.825	18.038	0.931
	HBR	92.400	13.792	
Stance Evaluation Weight Distribution (%) O	SVR	49.757	3.667	0.408
	HBR	48.870	3.371	
Stance Evaluation Weight Distribution (%) NO	SVR	50.243	3.667	0.408
	HBR	51.130	3.371	
Squat Analysis Average Weight Distribution (%) O	SVR	48.433	2.765	0.961
	HBR	48.395	2.338	
Squat Analysis Average Weight Distribution (%) NO	SVR	51.567	2.765	0.961
	HBR	51.605	2.338	

**Table 4:** Number of individuals returning to sport for the SVR and HBR groups, respectively. The table describes numbers and percentages of RTS to difference levels as reported in both the rehabilitation groups.

RTS	SVR Group	HBR Group
Same level	23 (76.6%)	16 (53.3%)
Lower level	5 (16.6%)	9 (30%)
No return	2 (6.6%)	5 (16.6%)

## Data Availability

The data presented in the study are available upon request from the corresponding author.
